# Mental health and school dropout across educational levels and genders: a 4.8-year follow-up study

**DOI:** 10.1186/s12889-016-3622-8

**Published:** 2016-09-15

**Authors:** Cathrine F. Hjorth, Line Bilgrav, Louise Sjørslev Frandsen, Charlotte Overgaard, Christian Torp-Pedersen, Berit Nielsen, Henrik Bøggild

**Affiliations:** 1 Public Health and Epidemiology Group, Department of Health Science and Technology, Aalborg University, Niels Jernes Vej 12, DK-9220 Aalborg East, Denmark; 2 Department of Clinical Epidemiology, Aalborg University Hospital, Sdr. Skovvej 15, DK-9000 Aalborg, Denmark

**Keywords:** Mental health, Gender differences, Education, Educational Dropout, Young Adults, Early School Leaving

## Abstract

**Background:**

Education is a key determinant of future employment and income prospects of young people. Poor mental health is common among young people and is related to risk of dropping out of school (dropout). Educational level and gender might play a role in the association, which remains to be studied.

**Methods:**

Mental health was measured in 3146 Danish inhabitants aged 16–29 years using the 12-Item Short-Form Health Survey and examined across genders and educational levels. For students, educational level at baseline was used; for young people who were not enrolled in school at baseline (non-students), the highest achieved educational level was used. The risk of dropout in students was investigated in administrative registers over a 4.8–year period (1^st^ March 2010–31^th^ December 2014). Odds ratios (OR) and 95 % confidence intervals (CI) were calculated for mental health and in relation to dropout in logistic regression models, adjusting for age, gender, educational level, parental education, parental income and ethnicity.

**Results:**

Poor mental health was present in 24 % (*n* = 753) of the participants, 29 % (*n* = 468) in females and 19 % (*n* = 285) in males (*p* < 0.0001). The prevalence differed from 19 to 39 % across educational levels (*p* < 0.0001). Females had a statistically significantly higher adjusted risk of poor mental health than males (OR = 1.8, CI = 1.5–2.2). Among the students the lowest risk was found at the elementary level (OR = 1.3, CI = 0.8–2.3), while students in higher education had a statistically significantly higher risk (OR = 1.9, CI = 1.2–2.9). The lowest-educated non-students had the highest OR of poor mental health (OR = 3.3, CI = 2.1–5.4). Dropout occurred in 8 % (*n* = 124) of the students. Poor mental health was associated to dropout in vocational (OR = 1.8, CI = 1.0–3.2) and higher education (OR = 2.0, CI = 1.0–4.2). For males in higher education, poor mental health was a predictor of dropout (OR = 5.2, CI = 1.6–17.3), which was not seen females in higher education (OR = 1.2, CI = 0.5–3.1).

**Conclusions:**

Poor mental health was significantly associated to dropout among students in vocational and higher education. Males in higher education had five times the risk of dropout when reporting poor mental health, while no such association was found for females.

**Electronic supplementary material:**

The online version of this article (doi:10.1186/s12889-016-3622-8) contains supplementary material, which is available to authorized users.

## Background

In the member countries of the Organisation for Economic Co-operation and Development (OECD), 20 % of young people end their education pathway before reaching upper secondary level, with potential negative consequences for both the individual and society [1–3]. This means that one in five students have a higher risk of facing unemployment, poverty, increased mortality and morbidity compared with their cohorts who complete their education [2, 4–6]. Mental disorders such as depression or anxiety increase the risk of dropping out of school (dropout) [3, 7–10]. However, not all mental health issues are diagnosed or classified as a mental disorder. Self-reported poor mental health is documented as particularly frequent among young females [11, 12] and may be associated to dropout.

The association between self-reported poor mental health and dropout should be evaluated in the context of other important factors. Socioeconomic status is one such well-known factor affecting dropout [13, 14] and the OECD recommends targeting policies towards economically disadvantaged individuals [1]. This is seen in current literature, where income of the parents and their educational background are being associated with poor educational attainment [15, 16]. Depression and anxiety increase the dropout risks for female students significantly [7, 17–19], which advocates for a focus on gender differences. However, despite the fact that females report poorer mental health than do males, female students are more likely to complete their education [20]. This seems paradoxical and indicates non-applicability of current knowledge within psychiatry when exploring self-reported poor mental health. Poor mental health is often considered as associated to dropout in lower educational programmes [2], but associations between mental health and dropout appear in higher education as well [21, 22]. Previous studies are restricted to cross-sectional data [3, 10, 21, 22] or examine dropout within a single educational level rather than comparing risks across levels [21–24]; therefore, possible differences in associations at different levels remains to be studied. A few longitudinal studies have examined the association between mental health issues in elementary school and dropout from subsequent educational levels [7, 17, 19]. However, this design is problematic because multiple events during educational transition may have an impact on both mental health and dropout. Present study examines potential differences in associations between mental health and dropout from on-going education across educational levels. The finding can be informative in future development of targeted prevention and treatment interventions.

We linked data on mental health from a representative sample of 3146 young survey participants to individual-level data from educational administrative registers to examine the prevalence and distribution of poor mental health across educational activities and genders. Further, prospective analyses of 1524 students were conducted to explore the extent to which mental health was associated with dropout and whether gender and educational level modified this relationship.

### Aim

The aim of this paper was (i) to describe the prevalence of poor mental health in young Danish people and (ii) to examine associations between poor mental health and dropout among students. Special attention was paid to potential gender differences and educational levels.

## Methods

The reporting adheres to the STrengthening the Reporting of OBservational studies in Epidemiology (STROBE) requirements [25].

### Study design and setting

The study was a cohort study performed among inhabitants in the North Denmark Region, one of the country’s five regions. Information on mental health was obtained from the North Denmark Region Health Survey in 2010, in which a stratified sample of 23,392 inhabitants over the age of 16 answered a postal questionnaire concerning health, morbidity and wellbeing. The response rate was 66 % for all age groups [11] and 58 % in the age group 16–29. Participants with an on-going education above elementary level (*n* = 1524) were followed in administrative registers during a 4.8–year period (1^st^ March 2010 – 31^th^ December 2014).

### Data sources

The survey information was linked to individual-level register data by use of the unique personal identification numbers of the participants [26]. We obtained gender, age and ethnicity from the Central Population Register, where all personal identification numbers of Danish citizens are listed with basic personal data [27]. The participants’ educational tracks were obtained from the Student Register, which holds data on every person who has attended educational programs authorised by the Danish Ministry of Education. All levels of on-going and completed education are registered with dates of enrolment, dropout or completion [28]. This register is reported by the Statistics Denmark as having a high reliability [29]. We tracked the personal identification numbers of the parents and collected information on parental education in the Population’s Education Register, a record of each person’s highest completed education. This register included records from immigration, enabling collection information on education completed outside Denmark. The register has a coverage of 96 % among the Danish Population and 85–90 % among the immigrant population [28]. Educational activities of the participants and parental education were managed according to UNESCO’s guidelines for classifying education, the International Standard Classification of Education 2011 (ISCED 2011). Parental income was collected from The Danish Income Statistics Register [30].

### Mental health

In the survey, the 12-Item Short-Form Health Survey (SF-12) constituted the mental health measure [31]. SF-12 is a generic measure shown useful in measuring physical and mental health even in smaller populations [32–34]. We managed the item scores in accordance with the User’s Manual for the SF-12v2® Health Survey [32] and calculated individual mental component summary scores (MCS-scores) ranging from 0 to 100. Higher scores indicate better mental health states. As recommended in the User’s Manual for the SF-12v2® Health Survey [32], a country-specific norm-based score was used to establish the cut-off point for having ‘poor mental health' [31–33]. The cut-off was set as the mean minus one standard deviation, corresponding to 44.3 when using the Danish normed score for the age group 18–44 [33]. All scores above this value were regarded as ‘good mental health’. Others have defined cut-off at the lowest 10^th^ [35] or 25^th^ [22, 36] percentiles of the MCS-scores in the study population. We ran sensitivity analyses using the lowest 10^th^ and 25^th^ percentiles in present sample.

### Dropout

Dates of dropout from the Student Register were used to identify dropouts within the follow-up period. We defined dropout as the event of leaving an education before completing the final exam. We only examined the education the students were attending at the time they answered the survey (on-going education). No non-students were included in the dropout examinations, although they may have enrolled in school during follow-up.

### Covariates

Age, parental education, parental income and ethnicity were included as covariates because they are anticipated to influence dropout [7, 15–18, 37, 38]. Baseline age of the participants was classified into three groups: 16–20, 21–25 and 26–29 years. Parental education was defined as the highest achieved educational level of the parents. The educational levels were defined as ‘Elementary school’ (ISCED levels 1–2), ‘Upper secondary education’ (ISCED levels 3–5) and ‘Higher education’ (ISCED levels 6–8). Parental income were defined by the highest income of the parents p.a. and categorized by sample tertiles and classified as ‘low’ (0–49550€), ‘middle’ (49551–67740 €) and ‘high’ (>67741 € p.a.). Data on ethnicity were dichotomized as’Ethnic Danes’ and ‘Non-ethnic Danes’. ‘Non-ethnic Danes’ included ‘Other Western’ and ‘Non-Western’ inhabitants [11].

### Educational level at baseline

**Fig. 1:**
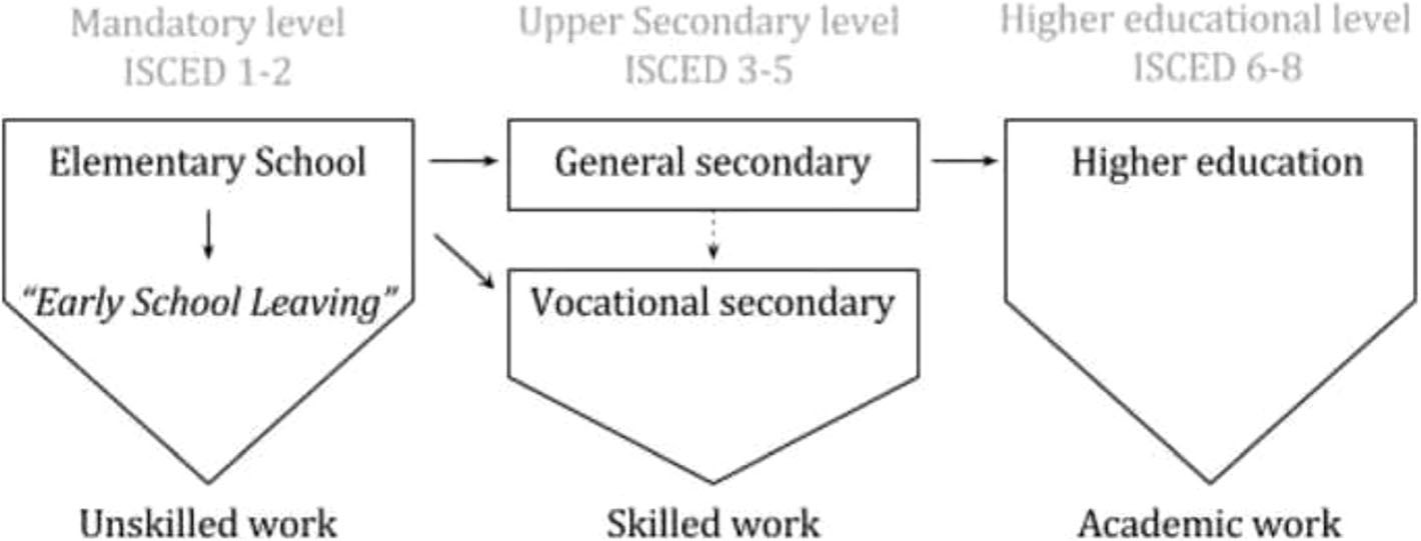
The normative educational pathway in the Danish school system. Elementary school is the only mandatory educational level in Denmark. This level is mandatory for 9 years, but a 10^th^ grade is optional and offered at some schools. Not entering upper secondary school is called “early school leaving”. This scenario may lead to employment in unskilled work. Upper secondary education can be either general or vocational (duration typically 1.5–3 years). The vocational secondary track qualifies one for skilled work, while the general track is compulsory before entering a higher education. A minor group (dashed line) enters the vocational secondary track after completing the general secondary level. Higher education qualifies one for academic work. Higher education can be accomplished by achieving a bachelor degree (duration 3–3.5 years), a master degree (duration further 2–2½ years) or a Ph.D. (duration further 3 years) [39]. The figure is normative, which means that some might choose non-traditional tracks, e.g. by entering unskilled work after completing general secondary instead of enrolling in higher education

Students were assigned into one of four groups by level of on-going education. The participants who were not pursuing an education at baseline were classified as non-students. The non-students were assigned into one of four groups by highest completed level of education at baseline. As illustrated in Fig. 1, the structure of the Danish school system was used to construct the educational groups and the participants were assigned in accordance with the ISCED level extracted from the registers [39].

As listed in Table 1, we divided the upper secondary level into the two tracks: vocational secondary and general secondary. Short cycle tertiary educations and vocational secondary educations primarily qualify for skilled work and were merged into one group. The remaining levels were listed as higher education [39, 40]. Early school leaving denotes a situation where a person does not enter or complete an upper secondary education after elementary school [1], which is not considered dropout according to the presented definition.

**Table 1:**
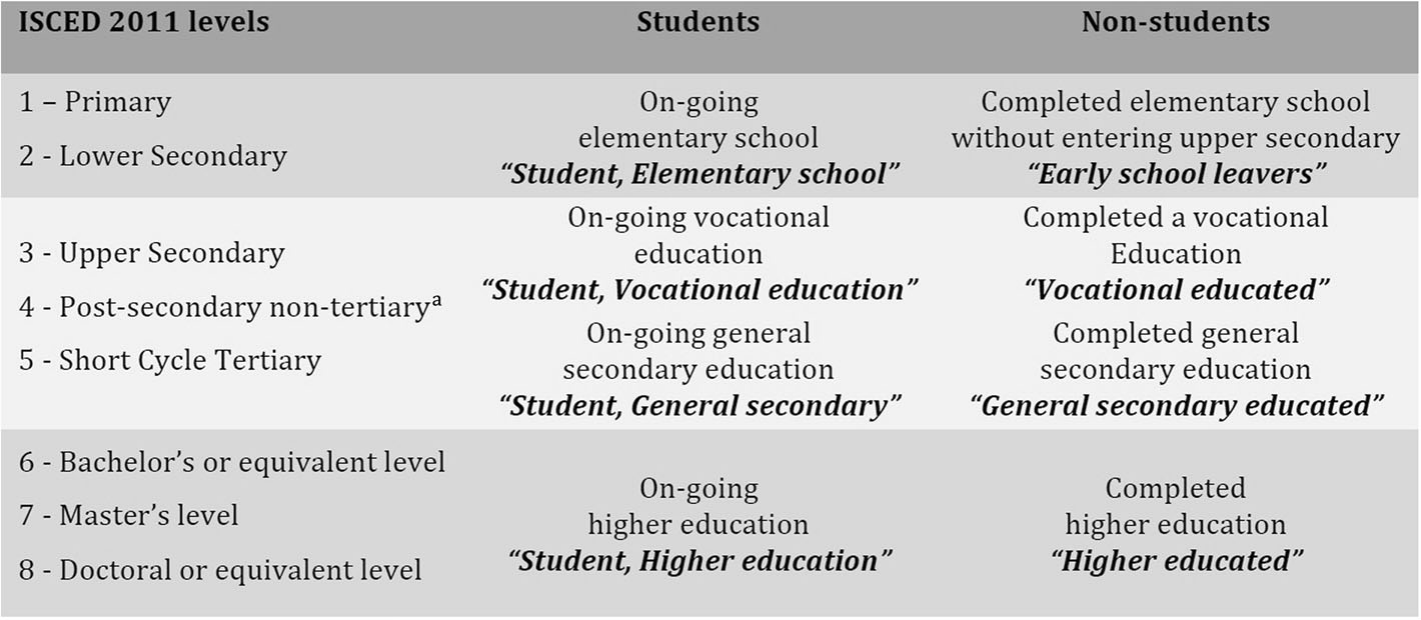
Educational group assignment of students and non-students

Participants were either students or non-students at baseline. Based on ISCED 2011 levels, the students were assigned into groups by on-going and the non-students by highest achieved educational level at baseline. The names of the educational levels are indicated in bold italic font

^a^: The post-secondary non-tertiary level does not exist in Denmark

### Selection of participants

**Fig. 2:**
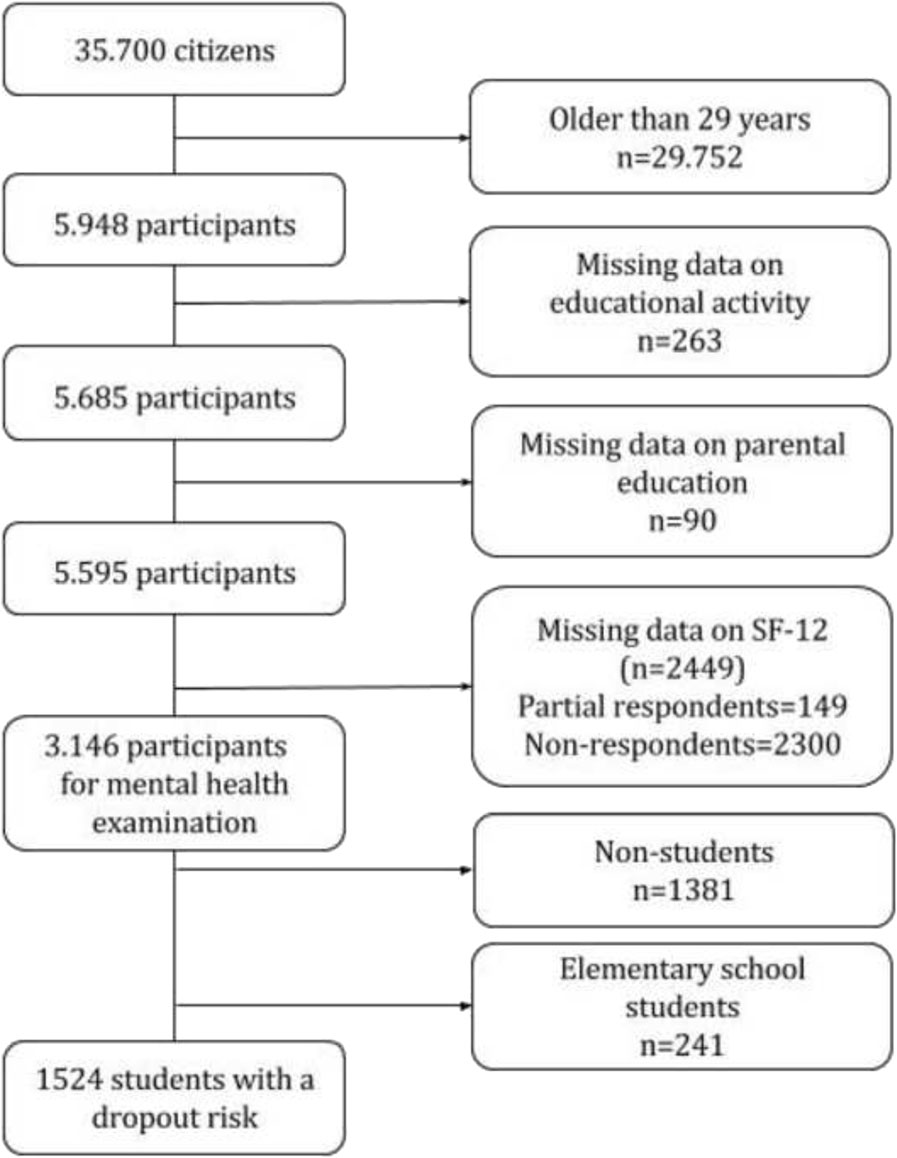
Flowchart of the selection of study participants. Right column shows the number (n) of participants excluded according to each criterion. 2300 of the citizens in the age group did not return the survey questionnaire and 149 did not answer all of the 12 items in the SF-12

The flow diagram in Fig. 2 illustrates the selection of the participants included in this study. The eligible participants were aged 16–29 at baseline, and we only analysed cases with complete data for all variables. Data of covariates were missing for some of the participants leading to exclusion. The outcome measure of mental health was missing for non-responders of the North Denmark Health Survey in 2010 and a few participants had only partial responses in the SF-12 questionnaire, leading to an uncalculated MCS-score. This left an effective sample of 3146 participants for the examination of poor mental health.

For the examination of dropout, we included students who at baseline were attending any educational level higher than elementary school, because attending elementary school is compulsory in Denmark and dropout is therefore extremely rare. This left 1524 participants with a dropout risk.

### Statistical analyses

Descriptive statistics of the participants were compared with *χ*^2^ statistics. To examine the extent to which educational level and gender were associated with mental health, we performed logistic regression analyses and calculated odds ratios (OR) and 95 % confidence intervals (CI). Second, the associations between mental health and dropout were studied in logistic regression analyses including design variables representing mental health at each educational level. All models were adjusted for age, gender, educational level, parental education and ethnicity. However, dropout analyses were stratified by gender. Interaction of sex and age were examined in the models by entering interaction terms. P-values below 0.05 were considered statistically significant. All analyses were weighted using SAS survey procedures (SAS® software, version 9.4; SAS Institute Inc., Cary, NC, USA) to adjust for unequal selection probabilities in the stratified sample. We conducted sensitivity analyses to investigate the consequences of changing the cut-off points for the dichotomization of the MCS-score in the regression models. Differences in descriptive statistics of non-responders and responders were tested with *χ*^2^ statistics.

### Ethical approval and consent to participate

All data were managed according to the World Medical Association’s Declaration of Helsinki [41], and the study was approved by the Danish Data Protection Agency (Ref.GEH-2014-014). The use of register-based data for scientific studies in Denmark requires no consent from the participants, nor permission from the National Committee on Health Research Ethics [26]. All data were managed and stored in servers held by Statistics Denmark, where encrypted identification numbers ensured a high level of protection of the participants’ privacy.

## Results

### Mental health at baseline

All selected demographic variables are presented in Table 2 and divided according to mental health status. A total of 753 participants (24 %) had poor mental health. Females numbered 468 (29 % of all the females) and males 285 (19 % of all the males). Among the students who dropped out 32 % reported poor mental health prior to their dropout. The proportion of people with poor mental health was highest in the oldest age group (26 %).

**Table 2:** Distributions of mental health across characteristics of the 16–29-year-old participants, with number of participants (n), column percentages (%) and Chi Square tests (*p*-value)

	Participants	Poor mental health	Good mental health	*P*-value
	n (%)	n (%)	n (%)	
Gender				
Females	1617 (51.4)	468 (62.1)	1149 (48.0)	<.0001
Males	1529 (48.6)	285 (37.9)	1244 (52.0)	
Age				
16–20	1293 (41.1)	299 (39.7)	994 (41.5)	0.4052
21–25	1009 (32.1)	238 (31.6)	771 (32.2)	
26–30	844 (26.8)	216 (28.7)	628 (26.2)	
Parental Education				
Elementary School	385 (12.2)	102 (13.6)	283 (11.8)	0.4218
Upper secondary	1619 (51.5)	386 (51.3)	1233 (51.5)	
Higher	1142 (36.3)	265 (35.2)	877 (36.7)	
Ethnicity				
Ethnic Danes	3077 (97.8)	730 (97.0)	2347 (98.1)	0.0643
Non-ethnic Danes	69 (2.2)	23 (3.0)	46 (1.9)	
Educational activities				
Student, Elementary school	241 (7.7)	48 (6.4)	193 (8.1)	<.0001
Student, Vocational secondary	508 (16.2)	99 (13.2)	409 (17.1)	
Student, General secondary	648 (20.6)	158 (21.0)	490 (20.5)	
Student, Higher education	368 (11.7)	100 (13.3)	268 (11.2)	
Early school leavers	204 (6.5)	79 (10.5)	125 (5.2)	
Vocational educated	557 (17.7)	127 (16.9)	430 (18.0)	
General secondary educated	389 (12.4)	97 (13.0)	292 (12.2)	
Higher educated	231 (7.3)	45 (6.0)	186 (7.8)	
Parental income				
Low	1048 (33.3)	296 (39.3)	752 (31.4)	0.0003
Middle	1048 (33.3)	226 (30.0)	822 (34.4)	
High	1050 (33.3)	231 (30.7)	819 (34.22)	
Dropout during follow up				
Yes	124 (8.1)	40 (11.2)	84 (7.2)	0.0154
No	1400 (91.9)	317 (88.8)	1083 (92.8)	

The gender difference in mental health was significant (*p* < 0.0001) and independent of whether the participants were students or not (*p* < 0.0001, results not shown). Age, parental educational level and ethnicity did not differ significantly between groups with poor and good mental health.

In all educational groups at least one in five had poor mental health. The prevalence was highest among early school leavers (39 %) and differed significantly from the other educational groups (*p* < 0.001). The dropout incidence was significantly higher among students with poor mental health (Table 2, *p* = 0.0154). The results are presented in Additional file 1: Figure S1.

Table 3 presents the OR for poor mental health across educational levels. The associations were amplified when controlling for gender and age (Model 1), which implies confounding by gender and age. The ORs for educational level was persistent or attenuated slightly after adjustment for parental education, parental income and ethnicity (Model 2). The ORs shows that neither parental education nor ethnicity was associated to poor mental health, but low parental income were associated to poor mental health. In model 2 the risk of poor mental health was higher in all educational groups compared with the reference, the higher-educated (OR = 1). Early school leavers had the highest risk of poor mental health (OR = 3.3, CI = 2.1–5.4). Among students the risk increased as educational level increased, and a significant association between poor mental health and educational level was found in higher education (OR = 1.9, CI = 1.2–2.9). Gender did not influence the association between mental health and educational level (*p* = 0.252), but the adjusted models showed that females were 83 % more likely than males to report poor mental health.

**Table 3:** Logistic regression analysis showing adjusted estimates of the associations between poor mental health and educational level (OR, 95 % CI)

	Unadjusted		Model 1		Model 2	
Educational level						
Students, Elementary school	1.0	(0.7–1.6)	1.4	(0.8–2.5)	1.3	(0.8–2.3)
Students, Vocational secondary	1.0	(0.7–1.5)	1.5	(1.0–2.4)	1.4	(0.9–2.3)
Students, General secondary	1.3	(0.9–1.9)	1.7	(1.1–2.7)	1.7	(1.0–2.7)
Students, Higher education	1.5	(1.0–2.3)	1.9	(1.2–2.9)	1.9	(1.2–2.9)
Early school leavers	2.6	(1.7–4.0)	3.6	(2.3–5.8)	3.3	(2.1–5.4)
Vocational educated	1.2	(0.8–1.8)	1.7	(1.1–2.5)	1.6	(1.1–2.4)
General secondary educated	1.4	(0.9–2.0)	1.7	(1.1–2.6)	1.7	(1.1–2.6)
Higher educated	1.0	Reference	1.0	Reference	1.0	Reference
Gender						
Females			1.8	(1.5–2.2)	1.8	(1.5–2.2)
Males			1.0	Reference	1.0	Reference
Age						
16–19			1.0	Reference	1.0	Reference
20–24			1.0	(0.7–1.3)	0.9	(0.7–1.2)
25–29			1.2	(0.9–1.7)	1.2	(0.9–1.6)
Parental education						
Elementary school					0.9	(0.7–1.3)
Upper secondary					1.0	(0.8–1.2)
Higher education					1.0	Reference
Parental Income						
Low					1.4	(1.2–1.7)
Middle					1.0	(0.8–1.2)
High					1.0	Reference
Ethnicity						
Ethnic Danes					1.0	Reference
Non-ethnic Danes					1.4	(0.8–2.4)

Model 1: Adjusted for gender and age group

Model 2: Adjusted for gender, age group, parental education, parental income and ethnicity

### Examination of dropout risk among students

During follow-up, 8 % (*n* = 124) of the students dropped out. Of these, one-third (*n* = 40) had poor mental health (Table 2). The cumulative incidence of dropout was highest in vocational education (14 %) and in higher education (10 %) and were statistically significantly more prevalent among students with poor mental health with significance in higher education (*p* = 0.0289). The results are presented in Additional file 2: Figure S2.

The logistic regression models illustrated that covariates confounded the difference in dropout risks among students with poor and good mental health, and that educational level mattered (Table 4). Poor mental health was associated to dropout in vocational education (OR = 1.8, CI = 1.0–3.2) and higher education (OR = 2.0, CI = 1.0–4.2), and overall, adjusted estimates showed that low parental income was significantly associated to dropout (OR = 2.9, CI = 1.7–4.7). Dropout did not differ significantly between genders or ethnic groups or among age groups. The stratified analyses suggest that in higher education, the association between poor mental health and dropout was stronger among male students (OR = 5.2, CI = 1.6–17.3) than female students (OR = 1.2, CI = 0.5–3.1).

**Table 4:** Logistic regression models estimating the associations (OR and 95 % CI) between mental health and dropout specifically for each educational level in the full sample and stratified by gender

	Full sample^a^		Females^b^		Males^b^	
	*n* = 1524		*n* = 829		*n* = 695	
Mental health						
Poor mental health / Vocational education	1.8	(1.0–3.2)	1.8	(0.8–4.4)	1.6	(0.7–3.7)
Poor mental health / General secondary	1.1	(0.3–3.6)	1.7	(0.4–7.7)	0.7	(0.1–6.0)
Poor mental health / Higher education	2.0	(1.0–4.2)	1.2	(0.5–3.1)	5.2	(1.6–17.3)
Good mental health / Same educational level	1.0	Reference	1.0	Reference	1.0	Reference
Educational level						
Vocational education	1.0	Reference	1.0	Reference	1.0	Reference
General secondary	0.2	(0.1–0.3)	0.1	(0.0–0.3)	0.2	(0.1–0.5)
Higher education	1.0	(0.6–2.0)	1.5	(0.6–3.4)	0.6	(0.2–1.7)
Age						
16–20	1.0	Reference	1.0	Reference	1.0	Reference
21–25	0.6	(0.3–1.0)	0.5	(0.2–1.1)	0.6	(0.3–1.2)
26–30	0.5	(0.3–1.1)	0.4	(0.1–1.1)	0.8	(0.3–2.1)
Parental education						
Elementary school	1.3	(0.7–2.7)	1.2	(0.5–3.4)	1.7	(0.6–4.2)
Upper secondary	0.9	(0.6–1.4)	0.9	(0.5–1.8)	0.9	(0.5–1.6)
Higher Education	1.0	Reference	1.0	Reference	1.0	Reference
Parental income						
Low	2.9	(1.7–4.7)	3.8	(1.7–8.3)	2.4	(1.2–4.8)
Middle	1.5	(0.9–2.5)	2.2	(1.0–5.0)	1.0	(0.5–2.0)
High	1.0	Reference	1.0	Reference	1.0	Reference
Gender						
Females	0.8	(0.5–1.2)				
Males	1.0	Reference				

The mental health variables represent estimated relative dropout risks associated with poor mental health relative to good mental at the given educational level

^a^Adjusted for educational level, gender, age, parental education, parental income and ethnicity (estimates not shown)

^b^Adjusted for educational level, age, parental education, parental income and ethnicity (estimates not shown)

### Sensitivity analysis

Changing the cut-off point to the lowest 25^th^ percentile of the MCS-scores increased the limit by 0.29 MCS point, which did not affect the results. Applying the lowest 10^th^ percentile to the models enlarged the gender difference in mental health, as females became more than twice as likely to report poor mental health than did males (OR = 2.3, CI = 1.8–3.0). The OR of poor mental health was higher in every educational level compared with the higher educated. The different OR among students was nearly equal, with OR = 2.9 for elementary students and OR = 2.7 (all significant) for the rest of the students and the differences among non-students increased.

The dropout relative risks increased in both vocational (OR = 2.9, CI = 1.3–6.4) and higher education (OR = 4.3, CI = 1.9–9.7). No significant interactions with gender were found in the dropout analyses, but the female students’ dropout risks changed, indicating that poor mental health was significantlyassociated to dropout invocational education (OR = 3.3, CI = 1.2–9.4). The dropout risk among males with poor mental health in higher educations increased to OR = 9.3 (CI = 2.4–36.5).

### Characteristics of non-responders

Non-responders of the North Denmark Health Survey aged 16–29 years old included a higher proportion of students and non-students from elementary school and vocational educations, compared to the survey respondents (*p* < .0001). Among non-respondents a higher proportion had parents with lower education and parents with low income (*p* < .0001). Non-respondents were more often males (*p* < .0001) and non-ethnic Danes (*p* < .0001) and were more likely to dropout (*p* < .0001). The results are presented in Additional file 3: Table S1.

## Discussion

This study examined the occurrence of poor mental health among young people and explored the differences across educational levels and genders. All students with an on-going education above elementary level were followed in educational registers over a 4.8-year period to explore whether poor mental health was associated to dropout.

### Key results

At least one out of five in every examined subgroup of young people reported poor mental health. We demonstrated that the relative risk of poor self-reported mental health was highest among early school leavers and that the relative risk increased with the level of education among the students. Females were significantly more likely to report poor mental health than were males. The risk of dropout differed across educational levels, with the highest levels seen among students undertaking vocational or higher education. In higher education, the male students with poor mental health had a five-fold greater risk of dropout than did those with good mental health. This association was not found for female students.

### Limitations

We acknowledge several limitations of this study. In the registers, self-determined dropout and school expulsion could not be distinguished, nor could we strictly distinguish dropouts from transfers to other forms of education. However, the timing of our data collection did limit the occurrence of transfers, because these mainly occur in the beginning of the school year, while the distribution of dropouts increases during the school year [14, 38]. The dropout prevalence of 20 % suggested by OECD was higher than the prevalence found in this study. This incongruence can be ascribed to OECD’s inclusion of transfers and early school leavers [1], and the lower prevalence in this study implies that we eliminated school leavers and transfers that, according to Tinto [14], would cause misleading classification when researching dropout related factors.

Non-respondents constitute a risk of selection bias because individuals with a low level of education in particular are less likely to participate in surveys [42, 43], which was also the case in this study. The differences between responders and non-responders in relation to gender and parental education might contribute to selection bias and might affect the results, as e.g. low parental education might have the effect that students have less social resources which possibly exposes them to dropout. However, non-responses are shown to bias prevalence estimates more than estimates of associations, which were our main aim to present [41–43]. A characteristic of non-respondents is found in Additional file 3: Table S1. The moderate sample size limited the power of this study, with the possible effect that the analyses were unable to show a statistically significant gender difference. We limited the sample sizes further to perform the gender-stratified analyses (despite the insignificant interaction) to explore potential subgroups’ risks as suggested by others [7, 17–19].

Parental education and parental income might act as proxies for underlying factors. Low parental income was associated to both poor mental health and dropout when having poor mental health among students. Such finding may reflect that some young people are affected by financial constrain [16], but as all Danish students have equal and free access to education and equal access to financial support during education (State Educational Grant) from the age 18 [44], this limits the confounding role of inequity in personal financial resources. Parental income may therefore reflect a social position of importance instead [45]. Parental education has been found to be associated with the expectations parents have for their children’s educations and the students’ persistence in education [14, 15, 46] and therefore may not solely reflect social position or economic resources. Thus, it is a strength that social position is incorporated, but it is possible that estimates could be confounded by other unmeasured economic or social determinants.

The questionnaire SF-12 has some limitations. Even though evidence supports that the MCS-score is a valid measure when studying psychiatric conditions as depression [47–49], bipolar disorders [50], anxiety [47] and severe mental illness [48] across groups in the general population, only moderate evidence are found for use regarding substance abuse and personality disorders [47]. However, the aim of this study was to examine associations between general self-reported mental health and dropout. Though, dichotomisation of the MCS-score challenges the opportunity to take the relative severity of the mental condition into consideration, as we cannot ensure the cut-off to be representative to the relative severity of the mental health. Neither is it possible to know if the cut-off point is equally representative for both genders, as no gender specific norms are calculated for a Danish population [33]. This is a limitation we will include in the following discussion.

### Strengths

The main strength of this study was the use of nationwide registers, providing complete follow-up and high validity in key information on covariates [26, 28, 29, 51]. In contrast with self-reported data, register data minimise bias in recall and social disability [51–53]. Furthermore, the registers enabled individual follow-up in this study, an advantage over some earlier studies using cross-sectional data [3, 10, 21, 22].

According to the World Health Organization, one aspect of mental health is that the”*individual realizes his or her own abilities*” [54]; this clearly suggests that the individual should play a role in determining his or her state of mental health. Thus, we believe it is an asset of the study that we derived mental health estimates from the well-validated and generic SF-12 questionnaire [31, 33, 34, 55] and used validated methods for interpretation, generating objective estimates of mental health [32]. Our sensitivity analyses demonstrated that the classification of poor mental health is important for these estimates.

### Overall mental health among young people

Gender differences in mental health seem to be a global challenge. Our findings were consistent with previous research concluding that mental health issues are more common among females [6, 13–15, 45, 46]. Gender differences are likely to be produced in a complex interplay of internal and external factors. Gender specific norms were calculated for SF-12 in the U.S, indicating a higher cut-off point for males [32]. No gender specific were calculated for a Danish Population [33]. Therefore, it should be taken into consideration, that females may report less severe mental health issues, whereas males may be reporting more significant distress.

Although social desirability bias is seen primarily in interviews with personal interaction [56] and we used a questionnaire validated for both genders [31, 32], male underreporting cannot be totally excluded. It is possible that males are more likely to adopt a stoic perspective and not reconcile themselves with responses implying weakness, which suggest an underestimation of poor mental health [57]. This may explain that males are more prone to dropout in higher education, because their reported mental health simply can be more serious than the females [32]. Thus, we may take the interpretation of the self-reported poor mental health among males into consideration in preventing dropout.

Young people have the highest unemployment rates [58], and such external factors can increase understanding of the higher risk of poor mental health seen among non-students. Early school leavers can be stigmatized when, from a community perspective, they are seen as disadvantaged in the labour market. Furthermore, those with lower education levels have poorer job possibilities, and these limited employment prospects can cause poor mental health [2]. The high unemployment rates might also help explain why poor mental health affects students in vocational and in higher education more than students in general secondary education, as the former types of students are about to enter the labour market with its discouraging employment prospects [58]. These conditions might cause competitive study environments and enlarge the perceived workload. According to Kausar [59] academic workload is a predictor of perceived stress among students, which also suggests that the positive gradient of risk by education level could be due to increasing levels of workload, especially in higher education.

### Student dropout risks

Gender differences found in studies of depression and anxiety might not be applicable to self-reported poor mental health conditions, as earlier studies found significant associations to dropout in females primarily [7, 17–19]. We found that females’ poor mental did not affect their risks of dropout. In contrast, male students might be in greater risk of dropout when their mental health is poor, especially students in higher education. This corroborates the gender difference found in higher education students in general [37, 38]. In a systematic review, Larsen et al. [38] found that male students had the highest dropout risk when transfers to other educations were excluded, but their study did not detect any evidence of the sources of the gender difference. Quinn suggests that, in contrast to female students, male students may have a lower level of discipline that could partially explain their increased risk of dropout [37]. Higher discipline levels in females possibly lead to greater persistence to achieve their career goals. Educational institutions and policy makers might also affect students’ responses to poor mental health. Poor mental health is a well-known problem among young females [11, 12], meaning that interventions have been primarily tailored to females, while the needs of male students may consequently have been overlooked. Males are less likely to seek counselling [60], which might be a key factor for understanding why poor mental health affects the dropout risks of males but not females. Females might be better at coping with poor mental health or recognizing they need to seek help.

Explaining differences in the associations between mental health and dropout across educational levels seems to be a complex endeavour, and dropout can presumably not be ascribed to personal factors alone. External factors may also play a role. We saw that mental health and dropout were associated in the work-qualifying vocational and higher education levels, but not in general secondary. This difference may be explained by the discouraging employment prospects the students in vocational and higher education are facing [61]. However, Tinto [14] argues that dropout is a process in which multiple factors interact; therefore, several external factors such as differences in school structures, resources and practices across the educational institutions might impact on dropout risks [1, 14, 38], and the differences across educational levels are still difficult to explain [17].

### Implications

It is widely shown that psychological counselling can prevent dropout among students [62–64], and that counselling centres are increasingly important in supporting students and their varying emotional needs [46, 65]. The implication of this study is that the self-reported mental health states of students are a potentially valuable factor for identification of those prone to dropout. Our results suggest that mental health issues should be taken into account when planning interventions aimed to prevent dropout, and that there may be a need to develop new interventions that are more attractive to male students because their help-seeking behaviour and needs might be different than those of females. Therefore, potential gender differences in counselling needs need to be explored further.

The strict classification of poor mental health in the sensitivity analysis suggested for both genders that the poorer mental health, the higher the dropout risk. This finding may justify stratified interventions where the needs of the worst-afflicted students are accommodated when resources must be prioritized.

## Conclusion

Poor mental health was a common condition among young people across all educational activities. Confounder controlled estimates showed that the risk of poor mental health was lowest among the young people who completed a higher educational programme. The early school leavers had the highest risk of all subgroups. Among students, the lowest risk was found among elementary school students and the risk was higher by each subsequent educational level. Overall, females were significantly more likely to report poor mental health than were males. The highest dropout risk associated with poor mental health occurred in students in vocational and higher education, with male students in higher education at highest risk. Analyses of females showed no significant associations between poor mental health and dropout.

## Additional files

Additional file 1: Figure S1. Prevalence of poor mental health as a percentage within each educational level. Bar charts presenting the prevalence of poor mental health across educational levels. Error bars represent 95 % confidence intervals. (DOCX 28 kb) Additional file 2: Figure S2. Dropout cumulative incidence as a percentage, among students with poor or good mental health during follow-up, by educational level. Bar charts demonstrates prevalence of dropout among students with poor mental health, compared to students with good mental health across educational levels. Error bars represent 95 % confidence intervals. P-values represent comparisons between poor and good mental health. (DOCX 30 kb) Additional file 3: Table S1. Analyses of non-respondents of the North Denmark Health Survey 2010, with number of participants (n), column percentages (%) and Chi Square tests (p-value). Table comparing characteristics of non-respondents with respondents of the North Denmark Health Survey 2010. (DOCX 15 kb)

## Abbreviations

CI: Confidence intervals
ISCED 2011: International Standard Classification of Education 2011
MCS-score: Mental component summary score
OECD: Organisation for Economic Co-operation and Development
OR: Odds ratio
SF-12: 12-item Short-Form Health Survey

## References

[1] OECD (2012). Equity and quality in Education: Supporting disadvantaged students and Schools.

[2] Patel V, Flisher AJ, Hetrick S, McGorry P (2007). Mental health of young people: a global public-health challenge. Lancet Lond. Engl.

[3] Lee S, Tsang A, Breslau J, Aguilar-Gaxiola S, Angermeyer M, Borges G (2009). Mental disorders and termination of education in high-income and low- and middle-income countries: epidemiological study. Br. J. Psychiatry..

[4] WHO. Mental health action plan 2013 - 2020. Geneva: 2013. http://www.who.int/mental_health/publications/action_plan/en/. Accessed 15 May 2015.

[5] Prince M, Patel V, Saxena S, Maj M, Maselko J, Phillips MR (2007). No health without mental health. The Lancet..

[6] Waghorn G, Saha S, Harvey C, Morgan VA, Waterreus A, Bush R (2012). “Earning and learning” in those with psychotic disorders: the second Australian national survey of psychosis. Aust. N. Z. J. Psychiatry..

[7] Fletcher JM (2008). Adolescent depression: diagnosis, treatment, and educational attainment. Health Econ.

[8] Roeser RW, Eccles JS, Strobel KR (1998). Linking the study of schooling and mental health: Selected issues and empirical illustrations at. Educ. Psychol..

[9] Veldman K, Bültmann U, Stewart RE, Ormel J, Verhulst FC, Reijneveld SA. Mental health problems and educational attainment in adolescence: 9-year follow-up of the TRAILS study. PLoS One. 2014;9(12):e115070. 10.1371/journal.pone.0101751 PMC4105412 25047692

[10] Borges G, Mora-Icaza MEM, Benjet C, Lee S, Lane M, Breslau J (2011). Influence of mental disorders on school dropout in Mexico. Rev. Panam. Salud Pública.

[11] Pedersen J, Friis K, Asferg AR, Hvidberg MF, Vinding AL, Jensen K (2011). Sundhedsprofil 2010 : trivsel, sundhed og sygdom i Nordjylland (The North Denmark Region Health Survey 2010).

[12] Illemann Christensen A, Davidsen M, Ekholm O, Pedersen PV, Juel K, Hvass LR (2014). Danskernes Sundhed : Den nationale sundhedsprofil 2013 (The Danish National Health Survey 2013).

[13] Rowan-Kenyon HT (2007). Predictors of Delayed College Enrollment and the Impact of Socioeconomic Status. J. High. Educ..

[14] Tinto V. Dropout from higher education: A theoretical synthesis of recent research. Rev Educ Res. 1975;45:89–125.

[15] Brown L, Iyengar S (2008). Parenting Styles: The Impact on Student Achievement. Marriage Fam. Rev.

[16] Chevalier A, Harmon C, Sullivan VO, Walker I (2013). The impact of parental income and education on the schooling of their children. IZA J. Labor Econ..

[17] Cornaglia F, Crivellaro E, McNally S. Mental Health and Education Decisions. London: CEE; 2012. http://cee.lse.ac.uk/ceedps/ceedp136.pdf. Accessed 15 July 2015.

[18] Needham BL (2009). Adolescent depressive symptomatology and young adult educational attainment: an examination of gender differences. J. Adolesc. Health.

[19] Sagatun A, Heyerdahl S, Wentzel-Larsen T, Lien L (2014). Mental health problems in the 10th grade and non-completion of upper secondary school: the mediating role of grades in a population-based longitudinal study. BMC Public Health..

[20] OECD (2014). Education at a glance 2014 : OECD indicators.

[21] Ruban PU, Petersen MG, Møller-Madsen B (2013). More than half of the medical students who apply for a dispensation drop out and need focused counselling. Dan. Med. J..

[22] Svensson AL, Strøyer J, Ebbehøj NE, Mortensen OS (2008). Factors predicting dropout in student nursing assistants. Occup. Med.

[23] Eisenberg D, Golberstein E, Hunt JB. Mental health and academic success in college. BE J Econ Anal Policy. 2009;9(1): Article 40.

[24] Ridder KAAD, Pape K, Johnsen R, Holmen TL, Westin S, Bjørngaard JH (2013). Adolescent Health and High School Dropout: A Prospective Cohort Study of 9000 Norwegian Adolescents (The Young-HUNT). PLoS ONE..

[25] ISBM University of Bern. STROBE Statement. Available checklists. http://www.strobe-statement.org/?id=available-checklists. Accessed 1 Apr 2015.

[26] Thygesen LC, Daasnes C, Thaulow I, Bronnum-Hansen H (2011). Introduction to Danish (nationwide) registers on health and social issues: structure, access, legislation, and archiving. Scand. J. Public Health..

[27] Pedersen CB (2011). The Danish Civil Registration System. Scand. J. Public Health..

[28] Jensen VM, Rasmussen AW (2011). Danish education registers. Scand. J. Public Health..

[29] Sørensen SM (2015). Documentation of statistics for The Student Registre.

[30] Baadsgaard M, Quitzau J (2011). Danish registers on personal income and transfer payments. Scand. J. Public Health..

[31] Jr JW, Kosinski M, Keller SD (1996). A 12-Item Short-Form Health Survey: construction of scales and preliminary tests of reliability and validity. Med. Care..

[32] Ware JE, Kosinski M, Turner-Bowker DM, Gandek B. User’s manual for the SF-12v2 Health Survey. Linc. RI Qual. Inc; 2007.

[33] Gandek B, Ware JE, Aaronson NK, Apolone G, Bjorner JB, Brazier JE (1998). Cross-validation of item selection and scoring for the SF-12 Health Survey in nine countries: results from the IQOLA Project. International Quality of Life Assessment. J. Clin. Epidemiol..

[34] Bjorner JB, Damsgaard MT, Watt T, Groenvold M (1998). Tests of data quality, scaling assumptions, and reliability of the Danish SF-36. J. Clin. Epidemiol.

[35] Illemann Christensen A, Kjøller M, Folker AP, Madsen LR, Nørgaard O, Hansen JA (2010). Mental sundhed blandt voksne danskere : analyser baseret på Sundheds- og sygelighedsundersøgelsen 2005 (Mental health among Danish adults: analysis based on The National Health Interview Survey 2005).

[36] Giver H, Faber A, Hannerz H, Strøyer J, Rugulies R (2010). Psychological well-being as a predictor of dropout among recently qualified Danish eldercare workers. Scand. J. Public Health.

[37] Quinn J. Drop-out and completion in higher education in Europe among students from under-represented groups. Eur Comm Netw Experts Soc Asp Educ Train NESET Eur Union. 2013. http://nesetweb.eu/wp-content/uploads/2015/09/2013-Drop-out-and-Completion-in-Higher-Education-in-Europe-among-students-from-under-represented-groups.pdf.

[38] Larsen MR, Sommersel HB, Larsen MS. Evidence on Dropout Phenomena at Universities. Danish Clearinghouse for educational research; 2013. http://forskningsbasen.deff.dk/Share.external?sp=Sdb267554-aa6f-4017-8e12-688a5b4f3c7c&sp=Sau. Accessed 6 July 2015.

[39] Ministry of Education. Overview of the Danish Education System. http://eng.uvm.dk/Education/General/Overview-of-the-Danish-Education-System. Accessed 28 July 2015.

[40] UNESCO Institute for Statistics (2012). International standard classification of education: ISCED 2011.

[41] World Medical Association. Declaration of Helsinki, ethical principles for medical research involving human subjects. JAMA. 2013;310(20):2191-4. doi:10.1001/jama.2013.281053. 10.1001/jama.2013.281053 24141714

[42] Christensen AI, Ekholm O, Kristensen PL, Larsen FB, Vinding AL, Glümer C, et al. The effect of multiple reminders on response patterns in a Danish health survey. Eur J Public Health. 2015;25(1):156-61. 10.1093/eurpub/cku057 24855288

[43] Anseel F, Lievens F, Schollaert E, Choragwicka B (2010). Response rates in organizational science, 1995–2008: A meta-analytic review and guidelines for survey researchers. J. Bus. Psychol..

[44] Danish Agency for Higher Education. State Educational Grant and Loan Scheme (SU). http://www.su.dk/english/state-educational-grant-and-loan-scheme-su/. Accessed 01 Apr 2016.

[45] Davis-Kean PE (2005). The Influence of Parent Education and Family Income on Child Achievement: The Indirect Role of Parental Expectations and the Home Environment. J. Fam. Psychol..

[46] Brunner JL, Wallace DL, Reymann LS, Sellers J-J, McCabe AG (2014). College Counseling Today: Contemporary Students and How Counseling Centers Meet Their Needs. J. Coll. Stud. Psychother..

[47] Andrews G, Henderson S, Hall W (2001). Prevalence, comorbidity, disability and service utilisation. Overview of the Australian National Mental Health Survey. Br. J. Psychiatry J. Ment. Sci.

[48] Salyers MP, Bosworth HB, Swanson JW, Lamb-Pagone J, Osher FC (2000). Reliability and validity of the SF-12 health survey among people with severe mental illness. Med. Care..

[49] Vilagut G, Forero CG, Pinto-Meza A, Haro JM, de Graaf R, Bruffaerts R (2013). The mental component of the short-form 12 health survey (SF-12) as a measure of depressive disorders in the general population: results with three alternative scoring methods. Value Health J. Int. Soc. Pharmacoeconomics Outcomes Res.

[50] Vojta C, Kinosian B, Glick H, Altshuler L, Bauer MS (2001). Self-reported quality of life across mood states in bipolar disorder. Compr. Psychiatry..

[51] Thygesen LC, Ersbøll AK (2014). When the entire population is the sample: strengths and limitations in register-based epidemiology. Eur. J. Epidemiol..

[52] Olsen J (2011). Register-based research: Some methodological considerations. Scand. J. Public Health..

[53] Hjollund NH, Larsen FB, Andersen JH (2007). Register-based follow-up of social benefits and other transfer payments: Accuracy and degree of completeness in a Danish interdepartmental administrative database compared with a population-based survey. Scand. J. Public Health.

[54] World Health Organization. Mental health: strengthening our response. 2015. http://www.who.int/mediacentre/factsheets/fs220/en/. Accessed 25 May 2015.

[55] Bjorner JB, Kreiner S, Ware JE, Damsgaard MT, Bech P (1998). Differential item functioning in the Danish translation of the SF-36. J. Clin. Epidemiol.

[56] Christensen AI, Ekholm O, Glümer C, Andreasen AH, Hvidberg MF, Kristensen PL (2012). The Danish National Health Survey 2010. Study design and respondent characteristics. Scand. J. Public Health.

[57] Fleishman JA, Lawrence WF (2003). Demographic variation in SF-12 scores: true differences or differential item functioning?. Med. Care..

[58] Scarpetta S, Sonnet A, Manfredi T (2010). Rising youth unemployment during the crisis.

[59] Kausar R (2010). Perceived stress, academic workloads and use of coping strategies by university students. J. Behav. Sci.

[60] Morgan NT, Robinson M (2003). Students’ help-seeking behaviours by gender, racial background, and student status. Can. J. Couns..

[61] OECD (2015). OECD Employment Outlook 2015.

[62] Sharkin BS (2004). College Counseling and Student Retention: Research Findings and Implications for Counseling Centers. J. Coll. Couns..

[63] Lee D, Olson EA, Locke B, Michelson ST, Odes E (2009). The Effects of College Counseling Services on Academic Performance and Retention. J. Coll. Stud. Dev..

[64] Turner AL, Berry TR (2000). Counseling center contributions to student retention and graduation: A longitudinal assessment. J. Coll. Stud. Dev..

[65] Mowbray CT, Megivern D, Mandiberg JM, Strauss S, Stein CH, Collins K (2006). Campus mental health services: Recommendations for change. Am. J. Orthopsychiatry..

