# Supplementary material for: Mental health and school dropout across educational levels and genders: a 4.8-year follow-up study
Source: BMC Public Health. 2016 Sep 15;16:976. doi: 10.1186/s12889-016-3622-8 (PMC5024430; doi:10.1186/s12889-016-3622-8)

Additional file 1

**Figure S1 - Prevalence of poor mental health as a percentage within each educational level.** Error bars represent 95% confidence intervals.


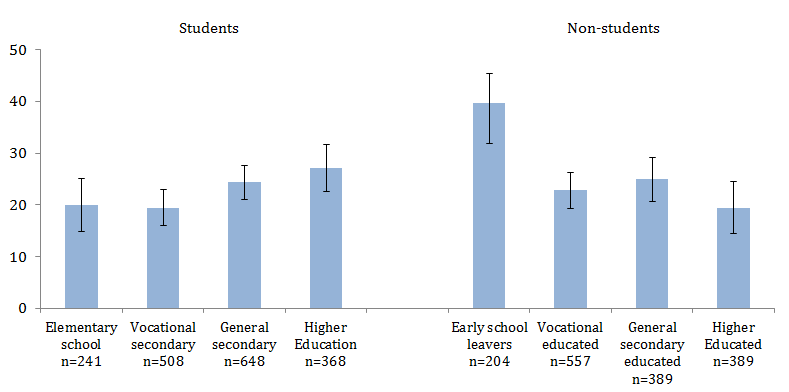

Supplement: Additional file 1: Figure S1. — Prevalence of poor mental health as a percentage within each educational level. Bar charts presenting the prevalence of poor mental health across educational levels. Error bars represent 95 % confidence intervals. (DOCX 28 kb) [file 12889_2016_3622_MOESM1_ESM.docx]
