# Supplementary material for: Mental health and school dropout across educational levels and genders: a 4.8-year follow-up study
Source: BMC Public Health. 2016 Sep 15;16:976. doi: 10.1186/s12889-016-3622-8 (PMC5024430; doi:10.1186/s12889-016-3622-8)

Additional file 2

**Figure S2 – Dropout cumulative incidence as a percentage, among students with poor or good mental health during follow-up, by educational level.** Error bars represent 95% confidence intervals. P-values represent comparisons between poor and good mental health.


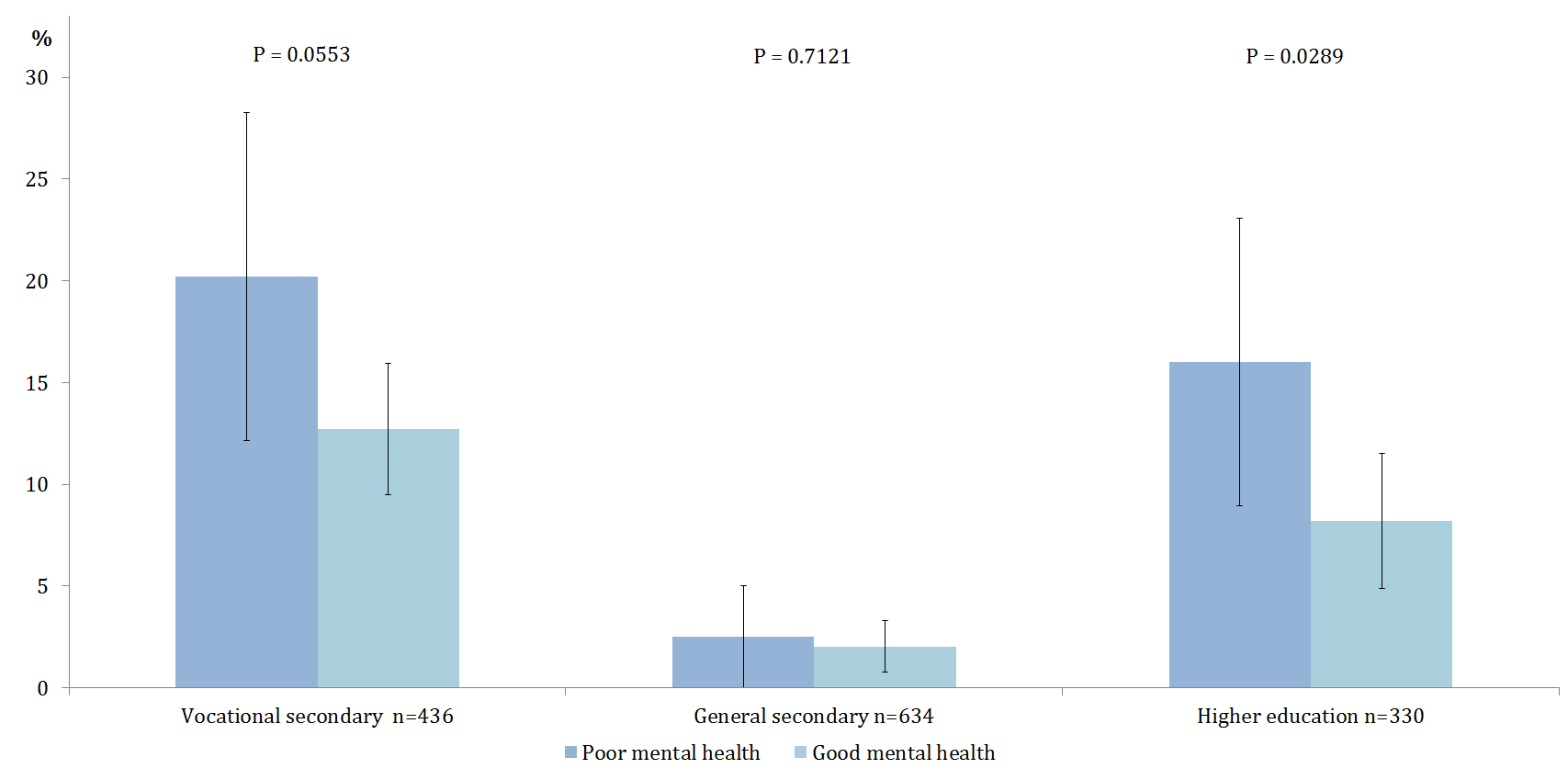

Supplement: Additional file 2: Figure S2. — Dropout cumulative incidence as a percentage, among students with poor or good mental health during follow-up, by educational level. Bar charts demonstrates prevalence of dropout among students with poor mental health, compared to students with good mental health across educational levels. Error bars represent 95 % confidence intervals. P-values represent comparisons between poor and good mental health. (DOCX 30 kb) [file 12889_2016_3622_MOESM2_ESM.docx]
