# Supplementary material for: Mental health and school dropout across educational levels and genders: a 4.8-year follow-up study
Source: BMC Public Health. 2016 Sep 15;16:976. doi: 10.1186/s12889-016-3622-8 (PMC5024430; doi:10.1186/s12889-016-3622-8)
Supplement: Additional file 3: Table S1. — Analyses of non-respondents of the North Denmark Health Survey 2010, with number of participants (n), column percentages (%) and Chi Square tests (p-value). Table comparing characteristics of non-respondents with respondents of the North Denmark Health Survey 2010. (DOCX 15 kb) [file 12889_2016_3622_MOESM3_ESM.docx]

**Additional file 3: Table S1 - Analyses of non-respondents of the North Denmark Health Survey 2010, with number of participants (n), column percentages (%) and Chi Square tests (p-value).**

| **n=5948** | **Respondents**  **n (%)** | **Non-respondents**  **n (%)** | **P-value** |
| --- | --- | --- | --- |
| Total | 3430 | 2518 |  |
| Gender | | | |
| *Females* | 1766 (51.5) | 997 (39.6) | <.0001 |
| *Males* | 1664 (48.5) | 1521 (60.4) |  |
| Educational level |  |  |  |
| *Students, Elementary school* | 274 (8.1) | 192 (8.4) | <.0001 |
| *Students, Vocational secondary* | 550 (16.3) | 484 (21) |  |
| *Students, General secondary* | 684 (21.2) | 266 (11.6) |  |
| *Students, Higher education* | 403 (11.9) | 174 (7.6) |  |
| *Early school leavers* | 225 (6.7) | 298 (12.9) |  |
| *Vocational educated* | 590 (17.4) | 563 (24.5) |  |
| *General secondary educated* | 412 (12.2) | 249 (10.1) |  |
| *Higher educated* | 247 (7.3) | 74 (3.2) |  |
| *Missing=263* |  |  |  |
| Parental Education | | | |
| *Elementary school* | 414 (12.6) | 382 (17.3) | <.0001 |
| *Upper secondary* | 1688 (51.2) | 1191 (54) |  |
| *Higher education* | 1198 (36.3) | 633 (28.7) |  |
| *Missing=442* |  |  |  |
| Parental income | | | |
| *Low* | 1239 (36.1) | 1210 (48.1) | <.0001 |
| *Middle* | 1094 (31.2) | 691 (27.4) |  |
| *High* | 1097 (32.0) | 617 (24.5) |  |
| Ethnicity | | | |
| *Ethnic Danes* | 3277 (95.5) | 2146 (85.3) | <.0001 |
| *Non-ethnic Danes* | 153 (4.5) | 369 (14.7) |  |
| *Missing=3* |  |  |  |
| Age | | | |
| *16-19* | 1394 (40.6) | 837 (33.2) | <.0001 |
| *20-24* | 1083 (31.6) | 1003 (39.8) |  |
| *25-29* | 953 (27.7) | 678 (26.9) |  |
| Dropout during follow up  (at risk=2561) | | | |
| *Yes* | 135 (8.3) | 135 (14.6) | <.0001 |
| *No* | 1502 (91.8) | 789 (85.4) |  |
